# Fabrication of Porous Alumina Structures by SPS and Carbon Sacrificial Template for Bone Regeneration

**DOI:** 10.3390/ma15051754

**Published:** 2022-02-25

**Authors:** Manuela González-Sánchez, Pedro Rivero-Antúnez, Rafael Cano-Crespo, Víctor Morales-Flórez

**Affiliations:** 1 Departamento Física de la Materia Condensada, Universidad de Sevilla, 41012 Sevilla, Spain; mgonzalezs@us.es (M.G.-S.); privero@us.es (P.R.-A.); racacres@us.es (R.C.-C.); 2 Instituto de Ciencia de Materiales de Sevilla, Centro Mixto CSIC-Universidad de Sevilla, 41092 Sevilla, Spain

**Keywords:** alumina, porous structure, mechanical properties, carbon, sacrificial template, spark plasma sintering, bone regeneration

## Abstract

In this work, a procedure for fabricating porous alumina with the use of a carbon sacrificial template has been tested in order to optimize the fabrication of porous structures mimicking the porosity and mechanical properties of the human cortical bone. Two different sources of sacrificial carbon were used and compared, and different sintering and calcination routes were considered. The porosity of the alumina structures studied by Hg porosimetry revealed that the amount of porosity and the size and shape of the pores are still below the required values, although some acicular pores were clearly observed by SEM. Moreover, measured mechanical properties (Young’s modulus) remained below that of the bone, suggesting the need for further consolidation treatments. In summary, these encouraging results drive the optimization of future fabrication routes.

## 1. Introduction

The current trend in the treatment of damaged tissues is the search for structures for which their objective is to regenerate tissues by itself while they perform its function. The multidisciplinary scientific field responsible for their design and fabrication is tissue engineering. This is based on three main concepts: cells, growth factors or physicochemical stimuli, and scaffolds [1,2,3,4], the object of interest in this study. Scaffolds are porous three-dimensional structures that provide a suitable environment for cells to proliferate and grow, thus forming new tissue. In order to select a material for the construction of a specific scaffold, a series of physical-chemical requirements should be fulfilled, and the different functions that will be performed when implanted in a specific tissue must also be taken into account.

In this study, the tissue that will be the focus of research is the cortical bone. Therefore, the requirements for bone regeneration materials are as follows [1,2,3,5,6]: biocompatibility, surface chemistry and topology that promotes cell adhesion, and highly porous structure with open and interconnected pores of adequate size (100–900 µm) [2,5,7,8]. The target porosity of the scaffolds must allow cell penetration, proliferation, and vascularization. Smaller sizes that may be present in the structure have other functions. In pores between 2 and 50 µm, there is no bone ingrowth [5]; in pores of 75–100 µm, there is ingrowth of unmineralized osteoid tissue; and the pores of 10–75 µm are penetrated by fibrous tissue and vascularization [9]. In addition, a porous structure will show a coarse surface that improves interfacial contact area between the biomaterial and the tissue, which promotes proper attachment of the implant and decreases its movement [10].

Currently, several groups are testing different materials for bone scaffolds, such as Liu et al. [11], who used a porous Nb-Ti-Ta alloy scaffold made by a combination of sponge impregnation and the sintering method. The obtained porous structures had a pore size in the range of 100–600 µm, a Young’s modulus between 0.11 and 2.08 GPa, and were suitable for apatite formation and cell adhesion. Moreover, Paredes et al. [12] developed calcium phosphate/polycaprolactone scaffolds by digital light processing (DLP) that had compressive strength above the range of cancellous bone. Finally, the work by Pei et al. [13] is also worthy of remark. They synthesized titanium porous scaffolds bioactivated by a coating layer of hydroxyapatite, exhibiting an elastic modulus of 3–5 GPa.

Alumina was chosen in this work as the material for the porous structure. For bone regeneration, alumina is a suitable candidate thanks to its biocompatibility and its excellent and tunable mechanical properties. In fact, alumina has already been considered as biomaterial for joint replacement and dental implants [14,15]. However, it is well known that alumina is not biodegradable; thus, permanent scaffolds (or implants) will be developed. The advantages of working with alumina-based permanent scaffolds rely on mechanical and structural stability throughout the entire regeneration process. Nevertheless, the main disadvantage of alumina is that it is bioinert (not bioactive), an undesirable characteristic for tissue engineering. Materials with bioactive surfaces are required because they favor cell growth and the bonds between tissue and biomaterial. Thus, some strategies have been proposed to bioactivate bioinert materials. For example, Esquivias et al. [16,17,18] showed that the addition of wollastonite conferred bioactivity to silica aerogels. Moreover, another alternative strategy has been proposed by Almeida et al. [19], who obtained bioactive hybrid materials made of PDMS-SiO_2_-TiO_2_-CaO by the sol-gel process, and the starting sol has been proposed as a coating to confer bioactivity to porous alumina [20].

In addition to bioactivity, cell adhesion, and porosity, it is highly recommended that the scaffold exhibits mechanical properties similar to those of the tissue to be reconstructed. This is particularly important for cortical bone, given the major role of its mechanical performance. Hence, a Young’s modulus similar to the cortical bone, namely, 7–30 GPa [21,22,23,24], is required for the scaffold, taking special care not to exceed this range of values in order to avoid stress shielding [21]. For this last reason, the use of hard ceramics for bone regeneration may be an interesting option instead of polymers or composites despite their lack of biodegradability, as the typical mechanical properties of polymer-based scaffolds are well below those of the cortical bone [9,24,25]. Moreover, material degradation of polymeric biodegradable scaffolds causes the weakening of the structure if the degradation rate is not adequate for the regeneration rate; thus, it can cause problems [2].

Regarding mechanical properties, bulk alumina exhibits excessive Young’s modulus (*E*_0_~400 GPa), much higher than that of the bone. This research pursues the exploitation of an insightful combination of the necessity of a porous structure and the relationship between Young’s modulus and porosity. It is well known that the Young’s modulus of the materials is highly sensitive to the microstructure of the material; that is, any defects and pores typically result in its reduction. Therefore, the required presence of porosity in the structure will involve a significant decrease in its mechanical properties in comparison to the bulk alumina. With this idea in mind, introducing a certain porosity to the alumina will reduce the Young’s modulus of the structure up to the range of the values of the cortical bone 7–30 GPa [21,22,23]. For this purpose, models that relate Young’s modulus and porosity (Table 1) are considered to estimate what porosity would be necessary to mimic the Young’s modulus of the bone.

**Figure 1:**
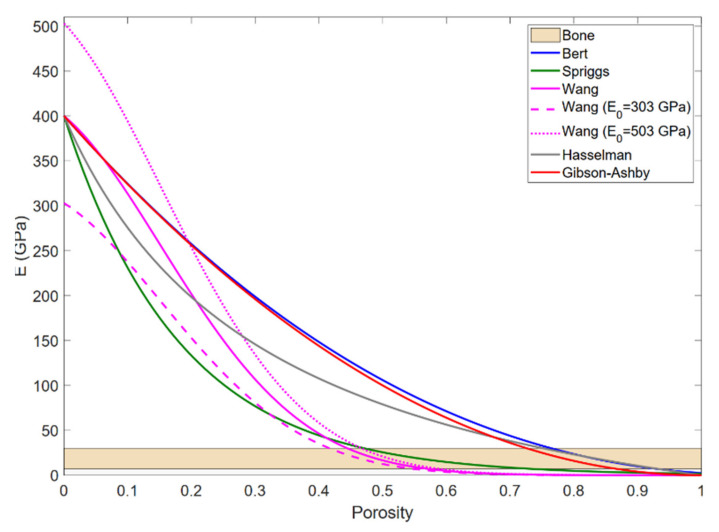
Dependence of the Young’s modulus of porous alumina, as a function of the porosity, regarding the indicated models. The considered Young’s modulus of the bulk alumina was *E_bulk-alumina_* = 400 GPa.

All models in Table 1 are empirical or semiempirical models satisfying evident boundary conditions, namely, for bulk alumina, *P* = 0, *E* = *E*_0_ = *E_bulk-alumina_*, and for very high porosity, *P* → 1, then *E* → 0 [26,33]. Figure 1 shows several models that could be candidates for simulating the system of interest, assuming typical *E_bulk-alumina_* = 400 GPa, and using parameters from the referred literature for alumina. In addition, in Figure 1, a reference horizontal band indicating the range of Young’s modulus of the bone has been included. The shape of the curve and the precise values where it cuts the reference band depend on the considered value of *E_bulk-alumina_*, as shown in the graph for Wang’s model.

The fabrication of porous alumina structures has been performed by different strategies such as the direct ink method [34], the gel-casting technique [34], freeze-casting [35], and the combination of spark plasma sintering (SPS) and sacrificial template employed by Choi et al. [36]. In this last case, these authors considered a sacrificial template procedure based on carbon. Hence, a carbonaceous phase was mixed with the alumina powder, and carbon was removed by calcination. To our knowledge, porous alumina structures have been never fabricated by SPS with bone regenerating purposes. This work has developed the Choi’s methodology by comparing different carbon phases as sacrificial templates and testing different fabrication procedures. The most suitable carbon phases and the preferable thermal and sintering routes are discussed in order to design an efficient fabrication procedure of alumina-based porous structures that emulate the porosity and mechanical properties of the human cortical bone.

## 2. Materials and Methods

### 2.1. Preparation of the Samples

The α-alumina powders (Nanostructured & Amorphous Materials Inc., Katy, TX, USA, 99% purity) were mixed manually in an agate mortar with the sacrificial template precursor powder (carbon). Two different sources of carbon were used: synthetic graphite powder (Alfa Aesar, Haverhill, MA, USA, 99% purity), labelled CG in this study, and charcoal activated for gas chromatography (Supelco-Merck, Darmstadt, Germany), labelled CM. The average particle sizes (Malvern Mastersizer 2000) of the powders are shown in Table 2. Carbon powders presented a monodisperse size distribution, and alumina powders presented a bimodal distribution.

**Figure 2:**
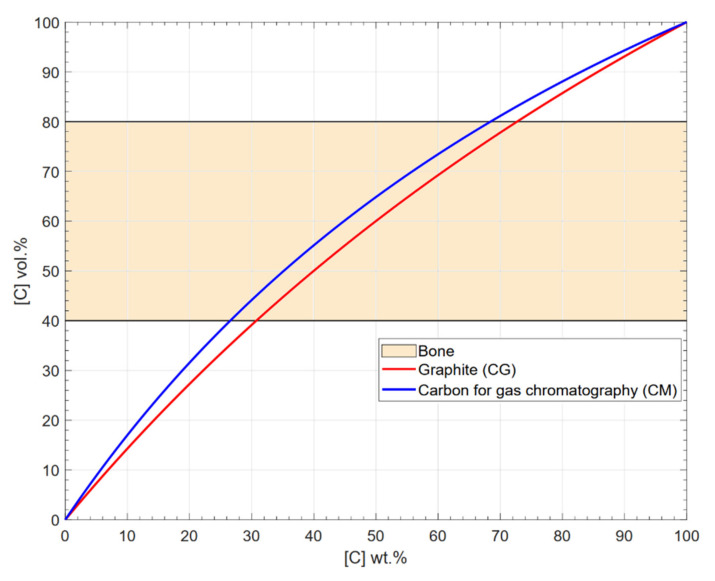
Relation between vol.% and wt.% for the carbon powders. The plot also shows the region of interest to possess porous alumina with Young’s modulus similar to the bone (labelled as ‘bone’ in the legend), regarding the empirical models cited in Section 1.

As a first consideration, the volume occupied by the carbon in the precursor powders is assumed to be the same porous volume in the final densified sample, although this assumption will be checked in this work. Thus, the carbon content was estimated from the target Young’s modulus of the porous structure of alumina. Then, the Young’s modulus of the porous alumina was determined by its porosity using the models from the Table 1 and Figure 1 by considering the extreme reported values of the Young’s modulus of the alumina: *E_min_* = 303 GPa and *E_max_* = 503 GPa. Hence, it can be verified that a porosity above 40% is necessary. Thus, the equivalent wt.% of required carbon can be estimated from the correlation between wt.% and vol.% shown in Figure 2, derived from the densities of bulk alumina (3.985 g/cm^3^) and carbon (CG density is 2.625 ± 0.003 g/cm^3^ and CM density is 2.1602 ± 0.0012 g/cm^3^, measured by Quantachrome device model Pentapycnometer 5200E). Therefore, a 40 wt.% of carbon was considered in this work, which is expected to confer an adequate porosity.

The samples were sintered by SPS in a Dr. Sinter Lab Inc. device, model 515S (Kanagawa, Japan), using the conditions from Table 3 for the different series of samples (S1, S2, and S3). The sintered disks were cut with a Bruker low speed saw ISOMET. Then, all samples were calcined in a muffle furnace at 900 °C for a dwell time of 5 h with heating ramp of 5 °C/min. This temperature has been selected after the results of thermogravimetric analyses (SETARAM Instrumentation, Caluire—France). Only the calcined S3 series received an additional heat treatment for 4 h at 1100, 1250, and 1300 °C and double heat treatment at 1250 and 1400 °C.

In summary, sample codes are *CG-SY-Z* and *CM-SY-Z*, where CG and CM stand for the origin of the carbon powder (graphitic carbon and carbon for chromatography, respectively), *SY* is the sintering conditions code regarding Table 3, and *Z* indicates the additional thermal treatment, if considered.

### 2.2. Characterization Techniques

The chemical composition and crystallographic structures of the samples were analyzed with an X-ray fluorescence (XRF) spectrometer (Panalytical model AXIOS) and X-ray diffraction (XRD), respectively. The diffraction experiments were conducted using a Bruker diffractometer model D8 advance A25 with a Cu anode, no monochromator, 40 kV, and 30 mA. The step-scanning technique ranged from 10° to 120° with a step of 0.015° and exposition time of 0.5 s.

For the study of microstructure, the samples were observed by scanning electron microscopy (SEM) and EDX; both techniques used a FEI model Teneo with acceleration voltages of 3 kV and 10 kV, respectively. Moreover, pore size distribution (PSD) was measured by using a mercury porosimeter. For this method, the Quantrachrome model Poromaster 60GT was employed.

Finally, the mechanical properties were studied with an INSTRON model 580, with a load cell of 5 kN and deformation rate of 0.5 mm/min. Loads were increased up to the fracture of the sample. For these tests, the samples were cut into parallelepipeds of squared sections with the following dimensions: 6 mm × 3 mm × 3 mm. Therefore, the standard expressed in ASTM D 7012-04 adapted to parallelepiped geometry of the aspect ratio height:width = 2:1 was fulfilled. Stress–strain curves were using the Bluehill INSTRON software by considering the geometry of the specimens. The Young’s modulus was estimated from the linear region of the obtained curves and averaged over three samples.

## 3. Results and Discussion

**Figure 3:**
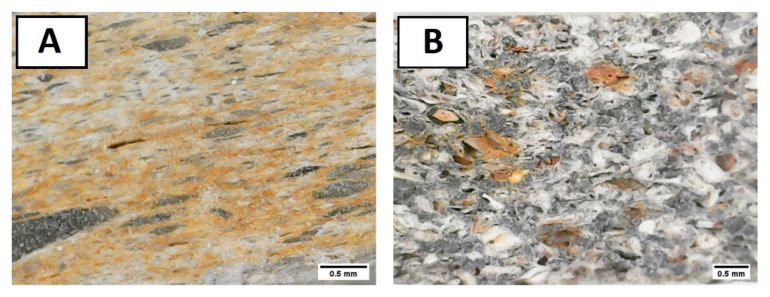
Optical images of CG-S3 (**A**) and CM-S3 (**B**). These images show, at macroscopic scale, the presence of some pores and impurities (gray and red spots) in the samples.

From these fabrication methods, porous alumina samples were obtained and subsequently characterized. A first exploration with a loupe at a mesoscopic scale revealed the presence of red and grey spots and pores with some preferential orientation (Figure 3). The images also show some isolated pores of ~100 µm.

Apparently, the gray spots may be due to remaining carbon, as not all of the sacrificial template was removed with the calcining routines or due to the diffusion of carbon through the alumina structure. In this regard, mass losses were estimated by measuring mass before and after the calcination of the carbon sacrificial template. Results indicated that CG lost all carbon (despite the presence of gray spots), whereas CM only removed approximately 36 wt.%. The chemical and crystallographic composition of the samples were studied to know the origin of impurities and colored spots.

### 3.1. Chemical Composition

The chemical composition of the resulting samples was checked by XRF, and the obtained compositions are shown in Table 4. The acquired results present high contents of Al and O consistent with an alumina precursor with the presence of oxide impurities (oxygen-aluminum molar ratio slightly higher than the stoichiometric ratio in pure alumina). Consistently, the results also show the presence of other elements and traces involving up to 7.8 wt.%. The significant number of impurities of Fe and Si must also be noted. While Fe impurities could have contributed to red spots on the samples observed after calcination and could be due to impurities of the carbonaceous phases, the origin of Si comes from the use of the charcoal activated for gas chromatography (CM powder).

In spite of the claimed purity of the precursors, these results are in coherence with the observed stained surface of the alumina samples and explain the incomplete mass loss observed by mass measurements during calcination of the carbon sacrificial template. Consequently, it may be taken into account that the calcination procedures tested in this work are slightly insufficient for the complete removal of the considered carbon phases. Nevertheless, these undesirable surface elements may be irrelevant thanks to the bioactivating coating that has to be applied to the structures in order to be used as bone regenerators. In addition, it is also known that the presence of different elements such as Si is highly recommended for improving cell adhesion and proliferation [37,38].

### 3.2. Structure of the Porous Alumina

**Figure 4:**
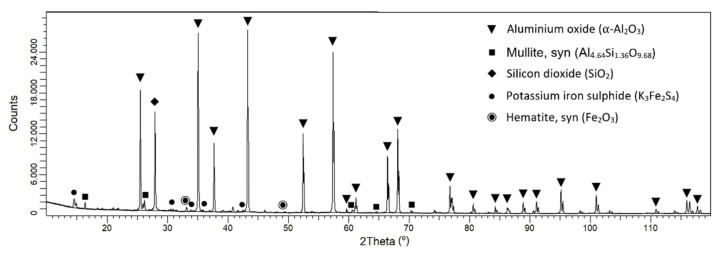
XRD pattern of sample CM-S3 where the main crystalline phases were identified as indicated in the legend.

The crystallographic composition of the obtained porous samples was revealed by XRD experiments. One representative diffraction pattern is shown in Figure 4, corresponding to sample CM-S3. It was confirmed that the stable alumina phase (PDF 01-075-1865), namely α-Al_2_O_3_, is the majority phase, as expected [39].

In addition, some of the impurities observed by the chemical analysis (Table 4) were identified as crystalline species indicated in Figure 4. Hence, Fe, S, and K are present as potassium iron sulfide (PDF 04-009-1496), and Si is forming aluminum silicates (PDF 01-079-1453) and silicon dioxide (PDF 01-082-1559). Fe is also present as hematite, which explains the red color of the spots that decorate the sample. The rest of the elements present in Table 4 could be forming amorphous phases or are in such small quantities that they were not observed by XRD diffraction.

**Figure 5:**
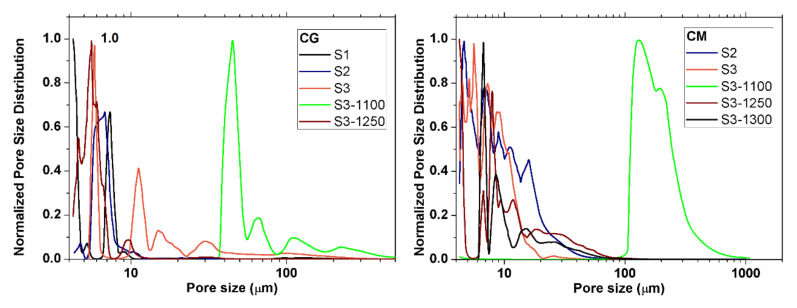
Pore size distribution of the CG and CM samples for different sintering conditions and heat treatments.

**Figure 6:**
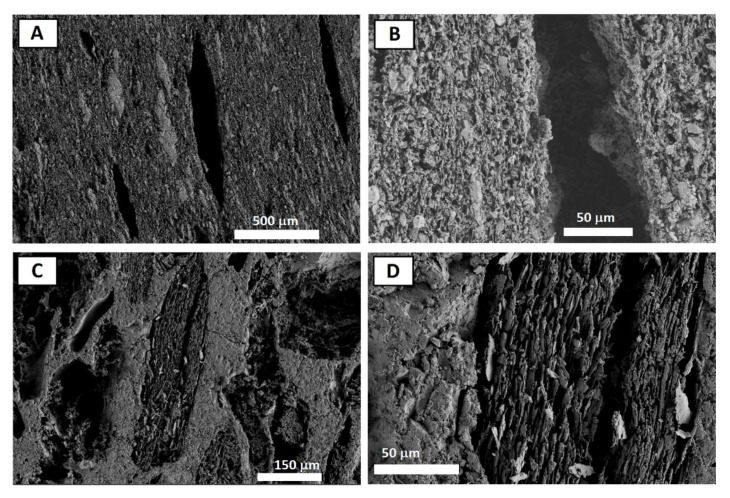
SEM images of CG-S3 (**A**,**B**) and CM-S3 (**C**,**D**).

Microstructural analysis was also performed by Hg porosimetry experiments. The normalized pore size distributions of the samples can be observed in Figure 5. Firstly, it should be noted the pores are significantly smaller than the sizes of the precursor powders. Hence, estimations and considerations about the quantity of the sacrificial template should be severely revised. This may be due to the pressure exerted during the sintering process. In almost all cases, all porosities were present below 20 µm. Pores with sizes below 4 µm are even present in sample CG-S1, as expected given its low heat treatment and also in sample CM-S3-1250. Almost all of pore sizes are far from the target minimum size of 100 µm, despite some pores of that size being observed in the loupe (Figure 3) and confirmed by SEM images (Figure 6). Only samples CG-S3-1100 and CM-S3-1100 have a very sharp peak at higher pore sizes, the former above 40 µm and the latter above 100 µm. Therefore, these two fabrication routes are encouraging procedures in terms of the resulting pore size distributions.

Regarding the total porosity of the samples, Hg porosimetry revealed values spanning from 6.9% for sample CM-S3 double heated up to 93.6% for sample CM-S3-1100, as shown in Table 5. Comparing data from samples S2 and S3 for the different carbon sources, the use of different carbon powders was relatively unimportant, as identical fabrication routes resulted in similar total porosities. The presence of dubious porosities higher than the expected 40% indicate that complete densification of the sample was not achieved and insufficient sintering occurred, and porosimetry experiments should further be performed in order to consolidate these results. In addition, the analyses of the porosities observed in the CM samples with increasing heating treatment confirmed the gradual occlusion of total porosity, although some erratic values were also obtained. Similar results were obtained in CG samples.

In addition, the direct inspection of the microstructure of the samples by SEM allowed for the identification of the pore sizes and shapes. In Figure 6, several representative images are shown. Figure 6A,B correspond to CG-S3 samples. They show isolated pores of the order of 100 µm with an acicular shape and preferential orientation perpendicular to axis of pressure applied during the sintering. On the other hand, Figure 6C,D are from CM-S3. These samples also have pores of the order of 100 µm on the surface with a slight preferential orientation.

In addition, in some pores, a laminar structure can be observed. There are two different types of skeletons of alumina in which one was completely densified and the other one was poorly densified. This imperfectly densified alumina can explain the high values of porosity observed by Hg porosimetry in these two samples (Table 5). Therefore, there are various structural features observed in SEM that match characteristics revealed by porosimetry. On the one hand, there is a predominance of small pores corresponding to those located in the laminar structure and the badly densified alumina; on the other hand, the low population of pores greater than 100 µm corresponds to the observed large acicular pores. Finally, the size of the pores is typically lower than the measured sizes of the original carbon powders (Table 2), especially in the case of CM samples. This can be explained in terms of a decrease in porosity due to the pressure exerted during the sintering process and due to heat treatment.

In summary, the presence of porosity in the alumina samples thanks to the calcination of the carbon sacrificial template was confirmed. However, although the existence of pores of adequate sizes (~100 µm) was confirmed, their relative presence in the sample is lower than the amount required for bone regenerating purposes. Nevertheless, these observed large pores may be interconnected by the small porosity revealed by Hg porosimetry.

### 3.3. Mechanical Properties

**Figure 7:**
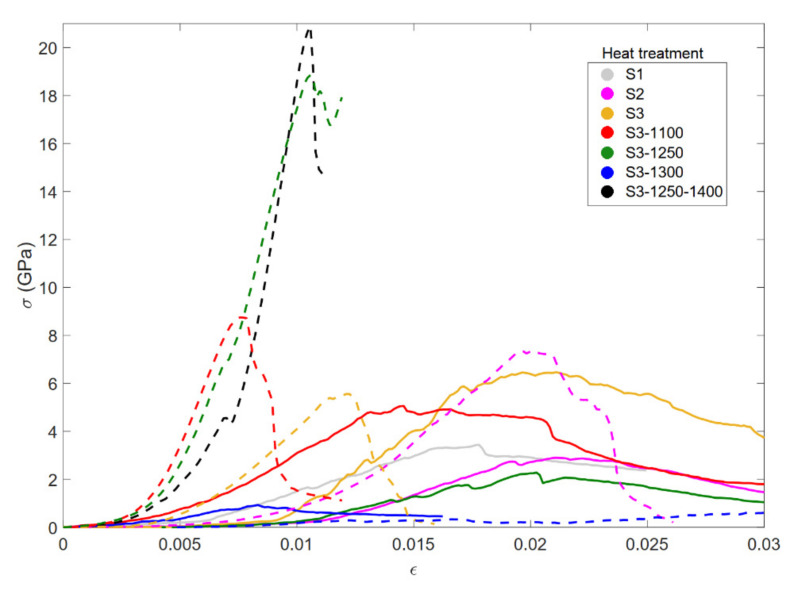
Stress-strain curves for CG (solid line) and CM (dashed line) samples. The colors correspond to different heat treatments.

The uniaxial compression tests allowed us to assess the mechanical performance of the porous alumina under stress. The stress-strain behavior of the samples is shown in Figure 7.

The different stress–strain curves plotted in Figure 7 indicate that CG samples exhibited a significantly softer response in comparison to the CM samples. This may be a consequence of the bad sintering of these samples, together with the observed porosity, that could also be even higher due to possible undetected small pores. Thus, the necessity of improving the sintering procedure of the CG sample series is concluded. On the contrary, CM sample series exhibited a quite reasonable mechanical response, increasing stress-at-fracture values with the increasing thermal treatment.

**Figure 8:**
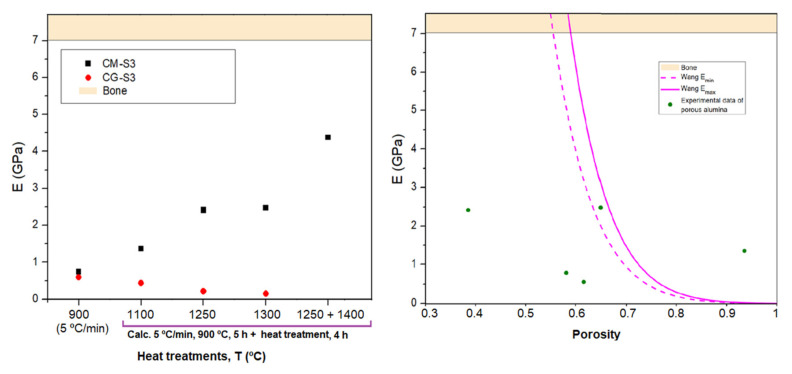
**Left**: values of Young’s moduli for samples CG-S3 and CM-S3. **Right**: dependence of the Young’s modulus with the porosity observed in the samples (dots) and corresponding models (lines). The reference values of the human cortical bone are highlighted as horizontally colored references.

The dependence of the Young’s moduli with the different carbon phases used as templates and with the different thermal treatments can be observed in data shown in Table 5 and Figure 8. Firstly, the inadequacy of the CG sample series is confirmed due to the very poor mechanical performance, which decreases with increasing thermal treatment. This strange behavior may be attributed to different porous structures that are formed with each carbon.

Regarding the CM sample series, there is an increasing trend of elastic modulus of CM with the temperature of heat treatment, as expected, and in coherence with the decrease in porosity (Figure 8, left). However, the maximum obtained value of Young’s modulus for the CM samples, 4.38 GPa for sample CM-S3-1250-1400, is still significantly lower that the lower limit of the cortical bone (7.5 GPa), but the evolution of the results is undoubtedly motivating. The relationship between Young’s modulus and the porosity of the CM samples is shown in Figure 8, right, and compared with two estimations of Wang’s model for minimum and maximum values of the elastic modulus of bulk alumina. The experimental values do not show a clear trend and are well distanced from the models. There is no well-defined relationship between porous structures and their Young’s moduli as a consequence of the problems in the consolidation of the alumina porous skeleton, the presence of badly sintered parts of the skeleton, and the probable presence of small porosity not revealed by low-pressure Hg porosimetry experiments.

## 4. Conclusions

This work has proven that two different carbon powders are suitable as sacrificial templates for the fabrication of porous alumina structures. Thus, with the aim of mimicking physical properties of the human cortical bone, porous alumina structures were synthesized in order to design the fabrication procedure of candidates for alumina-based implants. The starting consideration of volume-to-weight ratio resulted as not adequate due to the compression of the carbonaceous phase. In addition, incomplete densification was also observed in different samples; thus, the sintering process should be improved. Nevertheless, some of the used processes resulted in the presence of interesting pore sizes, namely, pore populations above 50 and 100 microns, which is very encouraging. Regarding mechanical properties, suggestive values close to 3 GPa measured in some of the structures with significant porosity indicate that high stiffness is being achieved for porous alumina, but those values are still quite below the target of 7 GPa of bone. Finally, due to the interesting results regarding porosity and mechanical properties, the optimization of the fabrication procedure is clearly encouraged, but the fabrication of porous alumina structures with suitable porosity and mechanical properties for emulating those of the human cortical bone is still a challenge.

## Figures and Tables

**Table 1:** Models of the dependence of the Young’s modulus of porous materials (*E*), with Young’s modulus of the bulk material (*E*_0_) and porosity (*P*). Each model considers different fitting constants and premises; see references for more details.

Model	Expression	Conditions	Refs.
Bert	$E=E_{0}{\left[1-\frac{P}{P_{max}}\right]}^{K_{0}P_{max}}$	Oriented porosity	[26]
Spriggs	$E=E_{0}\cdot e^{-bP}$	Low and moderate porosity, *p* ≤ 0.5	[27]
Wang	$E=E_{0}\cdot e^{-\left(bP+cP^{2}\right)}$	High porosity, *p* ≥ 0.5	[28,29]
Hasselman	$E=E_{0}\cdot \left[1+\frac{AP}{1-\left(A+1\right)P}\right]$	Loads parallel to the pores	[30]
Gibson-Ashby	$E=E_{0}{\left(1-P\right)}^{2}$	Open pores and isotropic material	[31,32]

**Table 2:** Particle size of powders. Uncertainties are one standard deviation of Gaussian fittings.

Powders	Particle Size (µm)
Alumina	1.58 ± 0.85 and 19.5 ± 8.7
CG	11.4 ± 4.2
CM	490 ± 130

**Table 3:** Sintering conditions for the different sample series.

Treatment Label	S1	S2	S3
Heating/cooling ramps	↑100 °C/min and ↓50 °C/min		
Plateau	1300 °C–5 min	1500 °C–5 min	
Uniaxial pressure (MPa)	75	50	75

**Table 4:** Results of XRF from the sample CM-S3 after calcination.

Element	wt.%	Element	wt.%
Al	44.60	P	0.14
Ca	0.48	S	0.17
Fe	0.32	Si	5.98
K	0.09	Sr	0.01
Mg	0.16	Ti	0.10
Na	0.31	W	0.03
Ni	0.02	Zr	0.01
O	47.58	-	-

**Table 5:** Porosity values estimated from tests of Hg porosimetry. Young’s moduli were assessed from uniaxial compression tests.

Sample Name		Porosity Hg (%)	Young’s Modulus (GPa)
CG	S1	91.0	0.302 ± 0.002
	S2	57.4	0.230 ± 0.005
	S3	68.3	0.529 ± 0.010
	S3-1100	32.0	0.429 ± 0.011
	S3-1250	32.5	0.216 ± 0.005
CM	S2	61.5	0.559 ± 0.012
	S3	58.1	0.792 ± 0.011
	S3-1100	93.6	1.36 ± 0.03
	S3-1250	38.52	2.42 ± 0.08
	S3-1300	6.9	2.48 ± 0.07
	S3-1250-1400	-	4.38 ± 0.10

## Data Availability

The data presented in this study are available in this article.
